# Dealing with the directive to restrict access to lethal means: parents’ perspectives

**DOI:** 10.1192/bjo.2025.10046

**Published:** 2025-06-27

**Authors:** Or Cohen Ben Simon, Yari Gvion, Shimrit Daches

**Affiliations:** Department of Psychology, Bar-Ilan University, Ramat Gan, Israel

**Keywords:** Suicidal behaviour, lethal means, parent, youth

## Abstract

**Background:**

Youth suicidal ideation and behaviour are major significant concerns, with suicide being the third leading cause of death among youth. In recent years, the trend toward deinstitutionalisation has caused parents of high-risk youth to face increasing emotional and practical challenges, including managing lethal means restriction (LMR) to reduce suicide risk.

**Aims:**

This qualitative study explores the experiences of parents instructed by mental health professionals to restrict their child’s access to lethal means in managing suicidal behaviours.

**Method:**

Twelve Israeli parents of youth aged 12–21 years participated in in-depth interviews. Using interpretative phenomenological analysis, the study investigated the emotional, psychological and relational challenges parents face when implementing LMR.

**Results:**

Findings indicate that parents struggle to understand and implement LMR guidance, experience emotional strain from their role as protectors, and face pervasive anxiety about their child’s safety. The study also highlights feelings of helplessness and the erosion of trust between parents and children. Many parents criticise LMR, viewing it as potentially harmful to their relationship with their child or ineffective at keeping their child safe.

**Conclusions:**

This study underscores the emotional and practical challenges parents face when implementing LMR. To improve its effectiveness, guidance should be re-evaluated and communicated more flexibly, emphasising shared responsibility between the parent and child, and address the emotional toll on parents during this critical period.

Youth suicidal ideation and behaviours are pressing concerns, with suicide ranking as the third leading cause of death among youth.^1^ Between 2016 and 2021, the mean annual incidence of emergency department visits and hospital admissions for suicidality in the USA increased significantly, highlighting a troubling trend.^2,3^ Parents of youth who have recently engaged in suicidal behaviour report symptoms of depression, anxiety, post-traumatic stress and stress-related issues such as sleep disturbances and weight loss.^4–7^ Deinstitutionalisation and earlier hospital discharges add further challenges for parents of high-risk youth, who often experience emotional and practical struggles related to their child’s mental illness.^8^

Despite these challenges, parents play a critical role in managing their child’s safety and recovery, including implementing lethal means restriction (LMR), a proven strategy to prevent or reduce suicide attempts by limiting access to lethal means such as firearms and medications.^9,10^ Studies show that when access to lethal methods is restricted, individuals are less likely to seek alternative methods and more likely to abandon suicidal intent.^11^ LMR counselling involves assessing access to lethal means and offering strategies to limit them, aiming to reduce opportunities for impulsive suicide attempts, which often occur during temporary crises.^12^ Most studies on LMR have focused on the effectiveness of tools and interventions in preventing access to lethal means and suicide risk,^13–15^ the attitudes and behaviours of emergency department providers (e.g. nurses and physicians) toward LMR counselling,^16^ and general LMR counselling practises.^17^

Although LMR serves an important preventive role, it can place considerable pressure on parents, who often become the primary guardians of their child’s safety. However, little is known about how parents experience the process of implementing LMR.

Qualitative data from two focus groups with parents of adolescents visiting the emergency room for suicide-related concerns revealed that many felt unprepared and overwhelmed when instructed to ‘keep an eye’ on their child and limit access to lethal means.^18^ Similarly, Ngwane and van der Wath^6^ conducted a qualitative study with ten parents, examining their psychosocial needs following their child’s suicide attempt. Some parents reported high levels of anxiety and described closely monitoring their child – even following them to the bathroom to prevent access to lethal means. Others described drastically altering their daily lives, constantly accompanying their child and feeling as though they were treating them as a ‘prisoner’. These findings suggest that LMR places significant stress on parents, yet it remains an understudied topic. Further research is necessary to gain better understanding of parental experiences and support needs.

## Aims

Because LMR is a well-established strategy that parents are encouraged to implement, it is crucial to understand parents’ experiences during LMR. Our study aims to answer several key research questions: (a) what are the emotional and relational impacts of LMR on parents? and (b) what challenges do parents encounter when implementing LMR and what are their needs?

## Method

Semi-structured interviews were conducted with parents who currently provide or have previously provided care for adolescents or young adults experiencing suicidal ideation and/or behaviour in Israel. Participation was voluntary, and written informed consent was obtained from all participants before the interviews. The authors assert that all procedures contributing to this work comply with the ethical standards of the relevant national and institutional committees on human experimentation and with the Helsinki Declaration of 1975, as revised in 2013.^19^ All procedures involving human patients were approved by the Research Ethics Board of Bar-Ilan University (approval number: 2023/32).

### Recruitment

Parents of youth and young adults (ages 12–21 years) who experienced suicidal ideation and/or behaviour were offered to participate through social media platforms. We focused on this age range because during this developmental stage, parents are typically the primary caregivers. Potential participants who reached out to the investigator received a telephone call from the research team, explaining the requirements and objectives of the current study. The research team scheduled a meeting with the parents who were interested in participating.

### Data collection

Parents were interviewed either in their homes, at a university laboratory or via Zoom, based on their preference. The interviews were conducted by the first author or by a graduate student in the clinical psychology track. Interviews lasted between 45 and 150 min, were audio-recorded and subsequently transcribed for analysis. A semi-structured interview format was used. The study was part of a large research project. Therefore, the current analysis was conducted on specific parts of the interviews that addressed the study questions. The full interview is available in Supplementary File 1 (available at https://doi.org/10.1192/bjo.2025.10046).

### Participants

Fourteen parents of youth and young adults (aged 12–21 years) who had experienced suicidal ideation and/or behaviour and were advised to restrict their child’s access to lethal means participated in the study (one parent per child). Because of technical issues, one interview was not recorded and thus excluded from analysis. Another parent decided not to participate because of a medical condition.

The final sample included 12 parents (11 mothers and one father) aged 39–58 years. Among them, ten were born in Israel, one in Russia and one in the USA. All participants identified as Jewish; eight described themselves as secular and four as Orthodox. Nine participants identified themselves as the primary caregiver for their child. Regarding the child’s gender, nine parents discussed their experiences with daughters, two with sons and one with a transgender son.

According to parents’ reports, eight children were diagnosed with borderline personality disorder. Other diagnoses were depression, bipolar disorder, obsessive–compulsive disorder, complex post-traumatic stress disorder, attention-deficit hyperactivity disorder and anxiety.

### Analysis

The data was analysed with interpretative phenomenological analysis (IPA),^20^ which focuses on understanding how individuals make sense of their experiences, emphasising subjective meaning. IPA employs a dual interpretation process (double hermeneutic), where participants interpret their experiences, and researchers analyse those interpretations through their own lens. IPA has been used in various qualitative studies, such as those focusing on adolescents’ self-harm^21^ and men’s experiences of suicide attempts and recovery.^22^

Analysis began with a detailed review of a single transcript, identifying themes by reading and rereading the text. Related themes were clustered, with some forming superordinate themes. The identified themes were cross-checked against the transcript for consistency with the participant’s words. Themes from the first transcript informed the analysis of subsequent ones while allowing new themes to emerge. Finally, a table of superordinate themes was created, capturing the key aspects of participants’ experiences and reflecting the interpretative process.

## Results

**Fig. 1:**
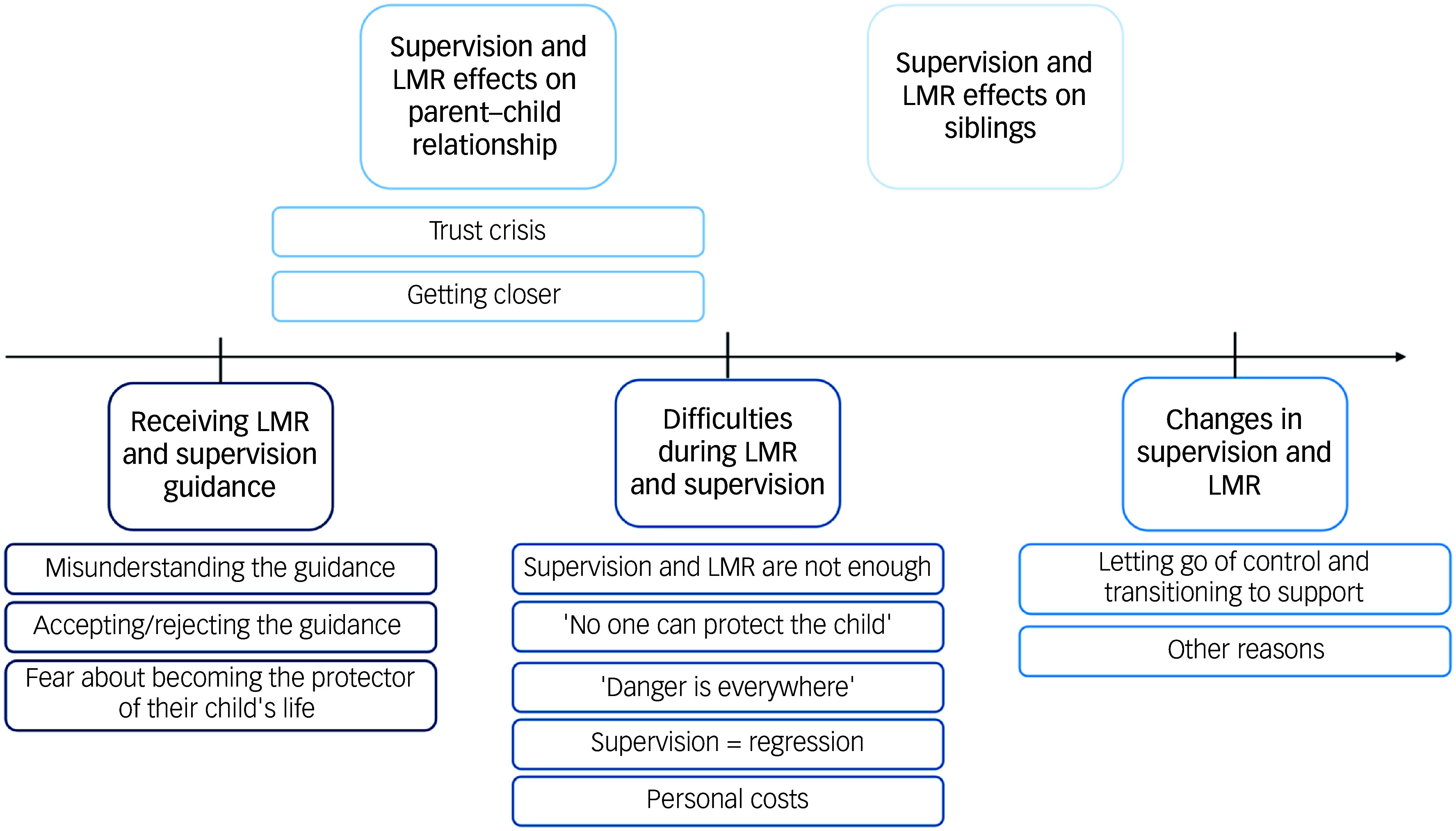
Main themes and subthemes results. Each colour represents a different theme. LMR, lethal means restriction.

The main themes and subthemes are summarised in the thematic map (Fig. 1).

### Receiving LMR and supervision guidance

Nine parents (75%) shared their experiences of receiving LMR and supervision guidance. Their responses fell into three subthemes: (a) misunderstanding the guidance, (b) accepting or rejecting the guidance and (c) feeling fear about becoming the protector of their child’s life.

#### Misunderstanding the guidance

Four parents (33%) reported that they did not fully understand the guidance; not comprehending the reason behind the guidance, nor how to implement it effectively. As a result, their actions to LMR were partial and inefficient:

*‘And then he told me, “always keep the psychiatric pills with you”. So, I carried a special bag to work where I could keep all the pills. And truly, they were with me wherever I went – back and forth – I didn’t leave a single pill at home. I have to point out that during that time, other medications were still accessible. I didn’t think about it at all – for example, she could have swallowed a lot of paracetamol or something like that. I just didn’t pay attention to other medications. And also, for example, knives – they stayed where they were; everything stayed in place’ (Carer 3).*

#### Accepting or rejecting the guidance

Five parents (41%) expressed their thoughts and feelings regarding LMR guidance, four parents expressed criticism or rejection of the guidance. Some expressed frustration with the difficulty of enforcing the guideline, highlighting practical barriers to removing dangerous items.

Parents experienced the guidance not only as unhelpful but also as harmful, as it involved intrusive behaviours toward the child (such as following them to the bathroom or asking them to bathe with the door open) and, in some cases, caused the child to use more harmful methods.

*‘During the home visits, this was the first time I was instructed on what to do. Not to leave her alone at all, she had to sleep in my bed, and if she went to the bathroom, the door had to be left open. I didn’t agree with that; I said there’s a limit to her invasion of privacy. What is she going to do in the bathroom in the five minutes she’s in there?’ (Carer 12).*

Some parents expressed concern that the instruction to implement LMR conveyed an unintended message to the child, implying a lack of trust in their ability to seek help independently.

Only one mother described a deep understanding of the guidance and its rationale, and felt that the guidance was reasonable and meaningful.

#### Feeling fear about becoming the protector of their child’s life

Six parents (50%) described feeling fearful about taking the responsibility of restricting access to lethal means and guarding their child to prevent suicide. Parents described fear and even terror that their child will die from suicide under their supervision, and felt afraid of being blamed if something were to happen.

*‘I was afraid she would do something more extreme than those cuts, and I also thought she’s in such distress, maybe a doctor should see her beyond that, maybe she needs hospitalisation… Why should I… I was afraid to take this on myself, that she’s at my house, under my responsibility’ (Carer 3).*

Parents also expressed that their fear led them to wish for their child to remain in hospital, and they felt pain and guilt for that. In some cases, parents even refused to take their child home from the hospital when the medical team wanted to release the child for a vacation. Some parents described the relief they felt when the child was admitted to hospital and someone else was guarding him/her.

### Parents’ difficulties during LMR and supervision

All parents (100%) described difficulties during LMR and supervision. Their responses can be categorised into five subthemes: (a) strict supervision and LMR are not enough, (b) feeling that ‘no one can protect the child’, (c) ‘Danger is everywhere’ fear that the child might die by suicide, (d) supervision equals regression and (e) personal costs.

#### Strict supervision and LMR are not enough

Seven parents (58%) shared that despite their strict supervision and diligent efforts, their child still managed to self-harm or attempt suicide. They described feelings of shock and disbelief upon discovering these incidents, with some expressing a sense of personal failure. Additionally, some parents reported feelings of anger toward their child, and frustration that their extensive emotional and practical efforts were not enough to keep their child safe.

*‘We were told from the start to remove knives and scissors. It didn’t help – she found ways. Whether it was taking the blade out of a pencil sharpener or using a compass, she found various methods that ended up causing even worse injuries, like carving. These alternative ways to hurt herself made it even more horrific. At one point, we even told her we’d bandage her hands, and then she started cutting herself on her stomach. She couldn’t stop – it was so intense. And throughout this whole time, there were no knives or scissors in the house; everything was locked away. She was constantly under supervision, yet she still managed to harm herself’ (Carer 2).*

#### Feeling that ‘no one can protect the child’

Many of the youth cycled in and out of hospital. Thus, the responsibility for LMR and safeguarding the child often shifted between parents and hospitals. Three parents (25%) expressed shock upon learning that their child had managed to engage in self-harm when under close supervision or had brought lethal means home from the hospital. These parents described feelings of helplessness and despair regarding their ability to protect their child, especially when a full medical team and a closely monitored institution were unable to do so. They also expressed disappointment with the medical institution for failing to ensure their child’s safety. Additionally, some parents voiced frustration with their child, feeling that their child was not trying to recover.

*‘The place operates at a very high level of supervision, meaning that as long as she was suicidal and harming herself, she was under 24/7 supervision by an aide. This means that even while she slept, someone was awake, sitting next to her. And yet, they still couldn’t stop the physical self-harm. They performed daily body checks on her; she had to stand in her underwear, and they would check what had changed from the previous day – every single day. They also thoroughly scanned all her belongings. Yet she still managed to hide blades and various tools, continuing to harm herself in a concealed manner’ (Carer 13).*

*‘He had two attempts during close hospitalisation… at two different hospitals… If you’re not keeping him safe, then what can I possibly do, for heaven’s sake? It’s such a feeling of helplessness’ (Carer 11).*

#### ’Danger is everywhere’, fear that the child might die by suicide

All parents (100%) described feeling constant vigilance, anxiety, and fear that their child might engage in deliberate self-harm or attempt suicide at any moment, even when supervised. Parents felt that danger was omnipresent and that fully restricting access to lethal means was impossible. Because many children hide their lethal means and suicidal intentions, parents described a loss of trust and the need to constantly examine their child’s belongings, repeatedly asking if their child was hiding something or had secret intentions to suicide.

Any separation from the child raised feelings of anxiety and fear that they might find their child injured or dead. Parents described feeling fear when they went to sleep, when the child took a bath, when their child didn’t answer their telephone or text messages, when they received a telephone call from their child, when the child travelled on the bus without them and when they returned home to re-meet their child.

*‘It’s scary, it’s stressful. I open the door to the house, and I don’t know what awaits me… I… to this day, I carry this feeling with me. On one hand, I don’t want to come home and see what’s happening there, but on the other hand, I just want to know that she’s okay’ (Carer 3).*

#### Supervision equals regression

Four parents (33%) emphasised that supervising their child is a manner appropriate for much younger children, but unsuitable for their child’s age and signified regression. One parent described that the constant need to supervise her teenage daughter as if she was much younger, led to feelings of anger, helplessness and despair. Another mother described the tension between supervision and respecting privacy, especially during adolescence, where there is a need to provide autonomy and allow the child to take care of themself.

#### Personal costs

Eight parents (66%) described the personal costs they had to bear when implementing LMR. Five parents (41%) mentioned that the requirement to supervise their child negatively affected their work or professional achievements, and in some cases, caused financial harm. Six parents (50%) shared their personal struggles, including a sense of loss of freedom, the constant need to choose between their own needs and their child’s, and giving up on activities like going to the gym or spend time with friends. Parents also described feelings of unhappiness because of the constant worry about their child, and anger toward their child when they had to cancel personal plans and activities.

*‘All the time having to choose between myself and her a little… For many years now, I feel like I don’t think about myself at all, only about her. Like I don’t even feel like I have anything I want anymore. It’s like everything has disappeared. What do I want? I don’t want anything. I just want everything at home to be okay’ (Carer 8).*

Some parents described feeling emotionally unavailable, worried, or guilty about going out, even when their child was in hospital and they no longer needed to restrict access to lethal means or guard their child.

### Changes in supervision and LMR

Eight parents (66%) expressed thoughts and feelings about reducing the level of LMR. Their responses can be grouped into two subthemes: (a) loosening control and transitioning to a supportive role, and (b) other reasons.

#### Loosening control and transitioning to support

Three parents (25%) described that at some point, they felt that supervision and LMR were impossible, unhelpful for their child and even harmful. As a result, they decided to decrease supervision and shift to a position of helping, aiding and supporting their child, knowing that their child would make their own choices for better or worse.

*‘And even more so, when she was looking for something sharp, I gave her a bowl of ice and a knife and told her, ‘First, try this’. Because the girl had completely lost control, and I couldn’t believe it. I thought, ‘It can’t be that I’m giving the child… I’m allowing her to have the knife’. Yes, she was just completely losing it, out of pain. I was afraid she might do something worse, and she tried to use the ice. The presence of the knife calmed her a bit… The knife was there so that if there was no choice, she could use it’ (Carer 2).*

#### Other reasons

Five parents (41%) described other reasons for reducing supervision and LMR. This included experiencing exhaustion and need for respite child supervision. In some instances, a reduction in the child’s suicide risk was reported, and another parent attributed the change to their child reaching 18 years of age.

*‘There were actually a few days, a week or so, without a suicide attempt. A week and a half – it was a lot compared to what… And we finally felt like we could breathe a little. Now we could go out for a night at a cabin’ (Carer 6).*

### Supervision and LMR effects on parent–child relationship

Five parents (41%) addressed the effect of their supervision and LMR on their relationship with their child. Although three parents (25%) described a trust crisis, two parents (16%) described that they were getting closer to their child as a result of their intense supervision.

#### Trust crisis

Although parents expressed a desire to communicate openly with their child and offer support when needed, they also described feelings of surprise and anger when confronted with their child lies. Parents described feeling hurt and devastated because of these lies and deceit. Some parents shared that the lack of trust created fear that, should their child genuinely need help in the future, they might not respond because of an inability to believe them.

*‘Listen, she’s so disrespectful, that girl. I sat with her, and she didn’t say anything to me about her struggles. I went upstairs to my room, and she took the pills. She didn’t even tell me – she sent… like, I went to the mall, and she asked me for an expensive perfume. I come back, she thanks me for the perfume, but she doesn’t say a word about the pills she took. I mean, there was a really deep breach of trust’ (Carer 3).*

#### Getting closer

Parents described how supervising their children brought them closer and, in a way, made them feel successful as parents despite all the criticism they had received.

*‘In hindsight, I see that suddenly I had success. Despite all the difficulty, I had a connection with my child that held him, in contrast to all the people who say, “You’re not a good mother, because of you he’s suicidal, you’re only harming your children, you’re just stressing them out”. Suddenly, I see that I’m close, that I’m close to my child’ (Carer 6).*

### Supervision and LMR effects on siblings

Five parents (41%) described how LMR affected the ill child’s sibling. Three parents (25%) mentioned that the other sibling experienced anger and frustration because of LMR, whereas two parents (16%) described how the sibling actively participated in the supervision effort and hid lethal means from their sibling.

*‘Because she was so aware, she would hide things herself that she thought could be dangerous for her sister’ (Carer 1).*

## Discussion

The current study explored parents’ experiences, challenges and needs during the process of receiving guidance and implementing LMR and supervision. The themes that emerged can be conceptualised around two central parts. The first is the timeline of parental experiences, which begins with receiving the guidance, moves through the difficulties encountered during the implementation process, and concludes with the potential reduction or alteration of LMR and supervision. The second addresses the impact of these guidelines on the parent–child relationship and the broader family dynamics, which was discussed both independently and through the timeline of parental experiences.

Study results indicated that approximately a third of parents misunderstood the guidance, leading to confusion and ineffective implementation. This may be partly attributable to variability in how LMR counselling is delivered, as previous studies have highlighted inconsistencies in communication practices.^23^ Alternatively, the high levels of distress experienced by parents during this initial period may have impaired their ability to process and fully comprehend medical guidance. Research among parents of children with medical illnesses has shown that parental distress negatively affects comprehension of medical information and decision-making.^24^ Furthermore, even when parents understood the guidance, many struggled to accept and implement it.

Results also revealed that half of the parents expressed fear about the responsibility of LMR and supervising their child, worrying about being blamed if something went wrong. This anxiety led some parents to wish for their child to remain in hospital. These findings align with a previous study that reported 57% of parents expressed a desire for their adolescent to remain in hospital following an emergency room visit for suicide-related concerns.^18^ We propose that this parental anxiety and fear of being blamed may reflect a form of projective identification with mental health professionals. Previous research has demonstrated that working with suicidal patients often elicits feelings of anxiety, panic, self-doubt and concerns about professional competence among mental health professionals.^25^ This distress, in some cases, reduced professionals’ willingness to treat suicidal patients and increased their tendency to refer such patients elsewhere.^26^ Professionals who deliver LMR may inadvertently transfer their anxiety and desire to shift responsibility to someone else, eventually leaving parents alone to manage their suicidal child.

Results also revealed that all parents experienced profound distress and anxiety throughout the process of LMR and supervision. In addition, strict supervision and efforts to limit access to lethal means were perceived by most parents as ineffective. A pervasive sense that ‘danger is everywhere’ emerged as a central theme and was associated with either chronic hypervigilance, repeated checks and heightened reactions or parental helplessness. In many cases, the supervision required parents to monitor and care for their adolescent child as if they were significantly younger, creating a sense of regression that conflicted with their expectations of fostering independence – a normative developmental milestone. Parents expressed shock, frustration and/or anger when their child managed to self-harm, whether at home under their supervision or during their hospital stay. Incidents at home were often internalised as personal failures, whereas events in medical settings led to disappointment and distrust in healthcare teams. These dynamics appear to have undermined parents’ self-efficacy and damaged both parent–child and sibling relationships. The shift in parental focus away from addressing their child’s emotional and developmental needs and fostering communication toward exerting control and surveillance proved ineffective at preventing self-harm and contributed to a loss of trust. The higher levels of parental distress, as well as the impact of LMR on siblings, suggest that therapeutic interventions for the entire family are necessary.

Parents reported (66%) that they decided to or thought about reducing LMR or supervision, which was sometimes driven by factors such as parental exhaustion or a reduction in their child’s suicidal risk. In other cases, LMR and supervision were reduced when parents decided to move from controlling their child’s behaviour – which was perceived as ineffective – to adopting a directive and supportive stance. This shift required parents to confront a profound and unsettling reality: the possibility that their child might engage in self-harm or even die from suicide, despite their best efforts. Parental roles are typically shaped by the imperative to protect children at all costs, making any form of ‘letting go’ deeply distressing. Accepting the risk of harm can feel counterintuitive and guilt-inducing, particularly when societal messages perpetuate the belief that suicide is always predictable and preventable – a perspective we know to be limited. Avoiding a rigid, black-and-white approach (accepting the guidance versus rejecting it) may help to address the complexities surrounding LMR and supervision. This calls for a re-evaluation of the guidance as well as how such guidance is delivered.

Based on parents’ reports regarding the perceived ineffectiveness of LMR, questions arise about the generalisability of LMR to all potentially dangerous items. Two prior large meta-analyses of suicide prevention strategies^9,10^ highlight that LMR strategies primarily focus on restricting access to firearms, pesticides (through regulation, content modification and secure storage), medications, methods of hanging, carbon monoxide, charcoal, barbiturate sales, caffeine tablet sales and implementing barriers at suicide hotspots; most of which have demonstrated effectiveness in reducing suicide rates. In contrast, removing knives or sharp objects, which is often recommended to parents, has received little empirical attention. For many families, removing sharp objects (e.g. scissors, razor blades) creates significant emotional and logistical challenges, often perceived as both stressful and ineffective. This finding aligns with prior research among adult patients, where difficulties in implementing LMR included challenges in LMR to household items like knives, which are used in daily life.^27^ Therefore, we propose re-evaluating the recommendation to remove knives and sharp objects from households. At the same time, we advocate for further research to examine the outcomes of such practices to ensure that LMR recommendations are evidence-based, effective and tailored to circumstances.

The parental conflict between safeguarding a child’s physical safety and fostering their emotional resilience – encouraging the child to take responsibility for their own life – highlights the need to rethink how LMR is communicated to families. This raises a key issue: should parents bear sole responsibility for restricting access to lethal means, or should the focus shift toward helping the child in taking responsibility for their own safety with appropriate parental support? Two recent systematic reviews examining the effectiveness of LMR counselling programmes reported that most youth-focused interventions target parents or caregivers.^17,28^ This approach may reinforce parental control while minimising or excluding the child from critical discussions about risk and responsibility. Just as clinicians involve adult patients in conversations about risks and safe management of lethal means, similar discussions should include youth, ideally in the presence of their parents.

Furthermore, because of the well-documented inconsistencies in delivering LMR,^23^ misunderstandings, confusion and ineffective implementation may occur. To address this, we emphasise the importance of LMR counselling for mental health professionals, to ensure that guidance is communicated clearly and accurately to both parents and youth. Previous studies have provided valuable insights into the rationale and effective implementation of LMR.^29,30^

Building on this knowledge^29,30^ and the current study’s findings, we recommend that clinicians first conduct a professional suicide risk assessment before initiating LMR counselling. This assessment should determine whether LMR is warranted, based on the patient’s current mental health status, history of suicidal ideation and behaviour, and relevant risk and protective factors. Once the need for LMR has been established, attention should shift to its effective implementation. Clinicians should assess specific environmental risks in the home, such as access to high places, sharp knives or highly lethal medications, and evaluate parental capacity to enforce restrictions. Rather than offering overly broad recommendations, clinicians should work with parents to identify the most relevant risks. It is essential that parents understand these distinctions and feel equipped to make informed decisions. Given the varying levels of parental anxiety, comprehension and ability to assess risks, clinicians should offer ongoing guidance and support throughout the LMR process.

Using a shared decision-making approach, the clinician, parent and adolescent should collaboratively develop an LMR plan tailored to the identified risks and circumstances. Each person’s role and responsibilities within the safety plan should be clearly defined. The rationale for LMR along with specific instructions should be provided in written form, and follow-up sessions should be conducted to evaluate the feasibility of implementation. In cases where parents experience difficulty distinguishing between high-risk and low-risk items or exhibit heightened anxiety, additional clinical support may be required to help them adopt a more balanced and practical approach to safety measures.

### Strengths and limitations

One main limitation of this study is its small sample size of 12 participants, which restricts generalisability. A larger, more diverse sample could provide additional insights. The participant pool was mostly mothers, with only one father, which may introduce gender bias. Future research should focus on capturing fathers’ perspectives and exploring differences between primary and secondary caregivers. Further, recruiting participants exclusively through social media may have introduced sampling bias, which could also limit the generalisability of our findings. Mental health diagnoses of children were based on parental report and were not confirmed by medical documentation. Additionally, since the study was conducted in Israel, where firearm possession is heavily restricted, the issue of firearm access was not addressed by most participants. This is particularly significant given that firearms are the most used method of suicide.^31^

Despite these limitations, the study’s strengths are notable. It is the first to examine parental experiences with LMR guidance, offering a detailed exploration of parents’ perspectives. The findings highlight challenges in LMR guidance and suggest the need for more tailored, effective and empathetic approaches in future interventions and research.

### Clinical implications

The findings from this study highlight the challenges parents face when implementing LMR and supervision, suggesting several clinical considerations. First, clear communication of LMR guidance is crucial, as misunderstandings were common and hindered effective implementation. Distress and anxiety among parents, which were exacerbated by perceived responsibility and fear of blame, need to be addressed in counselling sessions. Clinicians should recognise the impact of parental anxiety on their ability to support their child’s safety and autonomy. This highlights the need for strategies that empower parents and children. Furthermore, involving parents and children in counselling can improve engagement and ensure guidance aligns with family needs. Finally, ongoing research into LMR outcomes, particularly regarding household items like sharp objects, is crucial for effective, evidence-based interventions.

## Supporting information

Cohen Ben Simon et al. supplementary material Cohen Ben Simon et al. supplementary material

## Data Availability

Anonymised data is available from the corresponding author, O.C.B.S., upon reasonable request.

## Supplementary material

The supplementary material is available online at https://doi.org/10.1192/bjo.2025.10046
