# Supplementary material for: Dealing with the directive to restrict access to lethal means: parents’ perspectives
Source: BJPsych Open. 2025 Jun 27;11(4):e130. doi: 10.1192/bjo.2025.10046 (PMC12247063; doi:10.1192/bjo.2025.10046)
Supplement: Cohen Ben Simon et al. supplementary material [file S205647242510046Xsup001.docx]

*Semi-constructed interview –* The interviews included questions pertaining to two main domains:

**The impact of being instructed to limit child access to lethal means:**

1. What were your thoughts and feelings when you were told to limit access to lethal means?
2. What feelings does the instruction to limit access to lethal means provoke in you right now?
3. What influence, if any, has the instruction to limit access to lethal means had on your personal life?
4. What influence, if any, has the instruction to limit access to lethal means had on your relationship with your child?
5. What influence, if any, has the instruction to limit access to lethal means had on your family?
6. How do you think you will feel if you will be able to remove the restrictions of limited access due to your child feeling better?

**Identifying the parents’ needs in order to successfully restrict access to lethal means and protect their child:**

1. Did you implement the instruction given to limit access to lethal means? If not, why?
2. Can you identify factors that harm or assist your ability to follow through with the instruction given to limit access to lethal means?
3. What kind of help would you like to receive in order to successfully follow through with the instruction to limit your child’s access to lethal means?
